# Paranoid-halluzinatorische Psychose bei Intoxikation mit rotem Fliegenpilz

**DOI:** 10.1007/s00115-023-01511-6

**Published:** 2023-06-19

**Authors:** Michael Soyka

**Affiliations:** Psychiatrische Klinik München, Universität München, Nußbaumstr. 7, 80336 München, Deutschland

## Hintergrund

Pilzen kommt bei Intoxikationen eine große Bedeutung zu, wobei psychiatrische Aspekte oft kaum Erwähnung finden [21, 22].

Die Häufigkeit des Konsums halluzinogener Pilze in Europa ist noch niedrig mit einer 12-Monats-Prävalenz von maximal 1 % [2, 5, 8–11]. Für Deutschland wurde sie mit immerhin 0,5 % (Männer 0,7 %, Frauen 0,4 %) angegeben [20]. Gmel et al. [6] fanden für die Schweiz aber immerhin eine Lebenszeitprävalenz für Männer von 5,2 % (Frauen 2,2 %). Zunehmend bieten etwa Onlineshops z. B. psilocybinhaltige Pilze zu Aufzucht und Konsum an.

Meist handelt es sich bei Konsumenten um Personen mit experimentierfreudigem Probierverhalten. Physische Abhängigkeit und Entzug gibt es bei halluzinogenen Pilzen wie z. B. „magic mushrooms“ nicht [1, 2, 9–11, 14]. Die wissenschaftliche Literatur zu „magic mushrooms“ konzentriert sich fast völlig auf LSD bzw. psilocybinhaltige Pilze [1, 2, 7–11, 13], die auch mit Abstand am meisten konsumiert werden. Psychosen und auch Flashbacks können nach kurzem Konsum auftreten [14, 16]. Die Therapieforschung hat vor allem Psilocybin (wieder-)entdeckt [7]. Weniger bekannt ist, dass der in Europa heimische rote Fliegenpilz (*Amanita muscaria), *der zu den Isoxazolderivaten gehört, auch massive psychodelische Effekte hervorrufen kann. Die Substanz zählt zu den atypischen Halluzinogenen [16] und unterliegt nicht dem BTMG. Der Fliegenpilz ist kulturhistorisch interessant und ein populäres Motiv vor allem in Märchen, Sagen und Kinderbüchern [12, 16], wird aber bis heute bei schamanischen Ritualen genommen.

Als psychoaktiver Hauptwirkstoff gilt Ibotensäure und dessen Abbauprodukt Muscimol. Der genaue Wirkmechanismus ist nicht bekannt, gabaerge und serotonerge Effekte werden angenommen [11] Schwere Vergiftungen können auftreten [15, 19]. Psychotische Reaktionen sind bislang kaum beschrieben worden [3].

## Falldarstellung

Der 42-jährige Patient konsumierte seit der Jugendzeit Alkohol und Tabak, dann Cannabinoide, Ecstasy, später auch langjährig Heroin mit kurzer Substitutionsbehandlung, aber auch Halluzinogene. Die Familienanamnese bezüglich psychotischer Erkrankungen ist leer, der Proband litt auch nie an einer Psychose. Insgesamt zeigt der Patient ein experimentierfreudiges Suchtverhalten. Nach einer Haftstrafe und einer einmaligen Entwöhnungsbehandlung trinkt der Patient erhebliche Mengen Alkohol (5–10 Bier pro Tag) und konsumiert je nach Verfügbarkeit Cannabinoide, aber keine anderen illegalen Drogen mehr. Er hat eine Freundin, verbringt den Tag zu Hause mit Suchtmittelkonsum und Fernsehen.

Schon einmal nahm der Patient 4 selbst gesammelte Fliegenpilze. Ein mehrstündiger tranceähnlicher Rauschzustand, der als angenehm erlebt wird, wird berichtet. Einige Monate später sammelt der Patient ca. 10 große Fliegenpilze, kocht erneut einen Tee aus den Pilzen. Rasch entwickelt er lebhafte Wahnvorstellungen, leidet an optischen Halluzinationen und berichtet ein verändertes Zeiterleben. Eine Amnesie für das psychotische Erleben besteht später nicht. Der sehr drogenerfahrene Patient vergleicht die Wirkung am ehesten mit einem früheren Ketaminrausch. Der Proband erlebt sich und andere Mal als riesengroß, dann klitzeklein (Mikropsien), meint er sei ein Reiter in einer chinesischen Steppe. Stimmenhören wird nicht berichtet. Relevante somatische Symptome liegen nicht vor. Im weiteren Verlauf hat er starke Angst, ist erregt, schlägt auf die wohl als bedrohlich erlebte Freundin und Familienangehörige ein. Einige Stunden nach der Festnahme wird in einer Notfallambulanz eine Blutentnahme durchgeführt. Weitere therapeutische Interventionen erfolgen nicht. Später wird ein Ermittlungsverfahren wegen Körperverletzung eingeleitet.

Die Psychose klingt im Verlauf des nächsten Tages ohne Behandlung ab, relevante somatische oder neurologische Schäden liegen nicht vor. Eine Nachhallpsychose (Flashback) tritt nicht auf.

Die Drogenanalytik in einem akkreditierten forensisch-toxikologischen Labor zeigte eine niedrige Konzentration von Cannabis. Ibotensäure und Muscimol können aus analytischen Gründen auch in Speziallaboren nicht nachgewiesen werden.

Aus forensisch-psychiatrischer Sicht ist von einer substanzinduzierten Psychose (Intoxikation mit Wahrnehmungsstörungen), letztlich auch von einer Schuldunfähigkeit (Vollrausch) auszugehen.

## Diskussion

Der Konsum von Halluzinogenen, speziell den psilocybinhaltigen sog. „magic mushrooms“, ist kein Massenphänomen, aber auch nicht selten. Biodrogen bzw. halluzinogene Pilze kann man in Indolderivate (z. B. LSD und psilocybinhaltige Substanzen), andere pflanzliche Halluzinogene (z. B. Ibotensäure wie beim Fliegenpilz) und atypische Halluzinogene (z. B. Nachtschattengewächse) unterteilen [2, 11]. Die Prävalenz des Konsums von Halluzinogenen beträgt in Europa wohl < 1 % [5, 6, 8, 20].

Die Toxizität halluzinogener Pilze liegt weniger im somatischen [15, 17, 19] als im psychiatrischen Bereich. Als Komplikationen können auch nach einmaligem Konsum psychotische Rauschverläufe auftreten, ebenso wie Flashbacks bzw. (Nachhallpsychosen [16]).

Psychisch stehen Aufmerksamkeitsstörungen, Wahrnehmungsveränderungen, affektive Störungen, Beziehungsideen, paranoide Gedanken und Halluzinationen im Vordergrund. Möglicherweise können auch länger dauernde drogeninduzierte schizophreniforme Psychosen getriggert werden [2, 10–12].

Die meisten Studien an halluzinogenen Pilzen beziehen sich auf Indolethylamine (LSD) und Psilocybin, beides 5‑HT-2A-Rezeptor-Agonisten. Über die Toxizität von Fliegenpilzen ist weit weniger bekannt.

Fliegenpilze sind nach oralem Konsum wirksam mit einem relativ schnellen Wirkeintritt, z. T. innerhalb von Minuten [1]. Lange Zeit wurde vermutet, dass der Hauptwirkstoff Muscarin ist, ein Parasympathomimetikum, das im Körper zu einer Verlangsamung des Pulses, einer Verengung der Pupillen (Miosis) und einer Rötung der Haut durch Erweiterung der Blutgefäße sowie einer Erhöhung der Aktivität des Muskels des Magen-Darm-Trakts führt. Als Hauptwirkstoff wird heute eher Ibotensäure angesehen [15]. Beim Trocknen verwandelt sich Ibotensäure in Muscimol. Typisch ist beim Fliegenpilz eine Mydriasis, obwohl man bei Muscarin verengte Pupillen erwarten könnte.

Bereits 15 mg Muscimol können eine starke Intoxikation hervorrufen. Tödliche Vergiftungen sind sehr selten, Koma bei Intoxikationen wurde beschrieben [3, 19]. Ibotensäure und Muscimol wirken gabaerg [11], aber auch serotonerg und agonistisch am glutamatergen NMDA-Rezeptor.

Die berauschenden Effekte von Fliegenpilzen sind schon lange bekannt [13], möglicherweise reicht der schamanische Gebrauch von Fliegenpilzen bis in die Steinzeit zurück [18]. Psychopathologisch kommt es u. a. zu Psychosen, Deliren und schweren Intoxikationen. Die vegetativen Begleiterscheinungen können heftig sein. Generell können halluzinogene Pilze zu psychotischen Verläufen, Panik und auch sog. Horrortrips („bad trips“) führen. Flashbacks (Echopsychosen) oder andere psychiatrische Langzeitfolgen wie bei psilocybinhaltigen Pilzen sind bei Fliegenpilz nicht bekannt.

Christian Rätsch beschreibt die Symptome der Fliegenpilz-Berauschung als „parasympatholytische Erregung“ oder „toxische Ekstase“ [17], die im konkreten Fall aber eher einem „bad trip“ ähnelte. Der Konsum von Fliegenpilz ist häufig mit Visionen von Reisen in die Welt der Zwerge assoziiert ist [4], ähnlich wie im geschilderten Fall.

Zur Behandlung halluzinogen induzierter Psychosen liegen kaum Befunde vor [2, 11]. Evidenzbasierte Therapieempfehlungen existieren nicht. Der Einsatz von Neuroleptika wird nicht empfohlen, da darunter eine Exazerbation der Symptomatik wiederholt beschrieben wurde [11]. Die übrige Therapie ist symptomatisch, z. B. Benzodiazepine bei starker Angst und Erregung. Meist reicht es den Spontanverlauf abzuwarten.

Angesichts der relativen Offenheit mancher Konsumenten für neue Biodrogen ist nicht auszuschließen, dass in Zukunft auch vermehrt mit solchen Intoxikationen zu rechnen ist. Umso wichtiger ist die Kenntnis über die relative Gefährlichkeit entsprechender Konsummuster, zumal der analytische Nachweis sehr schwierig ist.
